# Prognostic Value of the Atherogenic Index of Plasma for Severity and Cardiovascular Outcomes in Young Patients (≤35 Years) with Coronary Artery Disease: A Retrospective Study

**DOI:** 10.3390/jcm15208092

**Published:** 2026-10-19

**Authors:** Jiayin Sun, Jiayin Liu, Yuning Wang, Fei Zhao, Wei Han, Yujie Zhou, Dongmei Shi

**Affiliations:** 1 Department of Cardiology, Beijing Anzhen Hospital, Capital Medical University, Beijing 100029, China; sjy616@163.com (J.S.); 15001326414@163.com (W.H.); azzyj12@163.com (Y.Z.); 2 Beijing Institute of Heart Lung and Blood Vessel Disease, Beijing 100029, China; 3 Beijing Key Laboratory of Precision Medicine of Coronary Atherosclerotic Disease, No. 2, Anzhen Road, Chaoyang District, Beijing 100029, China; 4 Beijing Anding Hospital, Capital Medical University, Beijing 100088, China; l13285346006@163.com (J.L.); m18345649667@163.com (F.Z.); 5 School of Public Health, Capital Medical University, Beijing 100069, China; halfanorangeee@gmail.com

**Keywords:** young patients, coronary artery disease, atherogenic index of plasma, major adverse cardiovascular events, coronary severity

## Abstract

**Background:** Rising coronary artery disease (CAD) in young adults calls for early risk stratification, yet the atherogenic index of plasma (AIP) has been understudied in patients aged ≤35 years. We therefore assessed its association with coronary lesion severity and major adverse cardiovascular events (MACE) in this cohort. **Methods:** This single-center retrospective study enrolled 948 patients aged 18–35 years with angiographically confirmed CAD. Greater coronary lesion severity was defined as a Gensini score > 40. The association between AIP and MACE was explored. **Results:** During a median follow-up of 60 months, MACE occurred in 287 patients. After full adjustment, AIP as a continuous variable was independently associated with greater coronary lesion severity (OR = 1.761; 95% CI: 1.052–2.948; *p* = 0.031). Regarding prognosis, when AIP was dichotomized at 0.4, it was independently associated with MACE (HR = 1.350; 95% CI: 1.060–1.721; *p* = 0.015) in the fully adjusted model, whereas continuous AIP did not retain significance. Restricted cubic spline analysis suggested a possible U-shaped dose–response relationship between AIP and MACE (overall *p* < 0.001, nonlinear *p* = 0.051). Subgroup analyses showed that the association of AIP with MACE was generally consistent across all examined strata, with no significant interactions (all *p* for interaction >0.05). **Conclusions:** AIP was independently associated with greater coronary lesion severity and MACE in young CAD patients (≤35 years). These findings support AIP as a practical tool not only for early risk stratification but also for guiding intensive lipid-lowering therapy in this distinct population.

## 1. Introduction

The proportion of coronary artery disease (CAD) in young adults is increasing annually, and acute coronary syndrome (ACS) is more commonly the initial manifestation, with approximately 4% to 10% of acute myocardial infarction (AMI) events occurring in individuals under 45 years of age [1]. A study involving 1013 young CAD patients aged 18–44 years reported that 45.3% were initially diagnosed with AMI, 54.0% had multivessel disease, and 19.2% were under 35 years of age [2]. Although young CAD patients have a relatively low early mortality rate, their long-term prognosis remains unfavorable [3]. According to statistics, approximately 50% of patients with premature CAD will experience substantial progression of coronary atherosclerosis within 10 years, and 20% will die prematurely [4]. A retrospective cohort study also showed that among young patients with a first-time diagnosis of CAD, 10% experienced myocardial infarction within 3 years after discharge [2]; moreover, a prospective cohort study revealed that one-third of premature CAD patients experienced an ischemic event, and 36% had at least one recurrent event [5]. Therefore, rapid assessment of the severity of coronary lesions and prediction of adverse events in young CAD patients are of great significance for their prognostic management.

Coronary atherosclerosis is a slowly progressive, multifactorial chronic inflammatory vascular disease. It is initiated by endothelial injury, which permits lipoproteins, particularly low-density lipoprotein (LDL), to infiltrate the subendothelial space. Following oxidative modification, these lipoproteins are taken up by macrophages in an unregulated manner, leading to foam cell formation, which in turn initiates and perpetuates the development of atherosclerotic plaques. Compared with larger LDL particles, smaller LDL particles are more prone to penetrate the endothelium, deposit in the arterial wall, resulting in enhanced atherogenicity [6]. The atherogenic index of plasma (AIP), a simple and readily available composite lipid biomarker derived from the ratio of triglycerides to high-density lipoprotein cholesterol (HDL-C), is strongly positively correlated with the cholesterol esterification rate and remnant lipoproteinemia, and inversely correlated with LDL particle size. It has been recognized as a potential independent risk factor for CAD [7]. Higher AIP levels have been associated with coronary artery calcification, multivessel disease, and an increased risk of plaque progression [8,9]. Moreover, elevated AIP is significantly associated with a higher risk of major adverse cardiovascular events (MACE), a finding consistently observed in both ACS and stable CAD populations [10,11]. In young patients with atherosclerotic myocardial infarction, dyslipidemia is typically characterized by reduced HDL-C and elevated non-HDL-C and triglycerides [12], supporting the potential predictive value of AIP in this population. Therefore, we aimed to investigate the association of AIP with coronary artery disease severity and its prognostic value in young CAD patients (≤35 years of age).

## 2. Materials and Methods

### 2.1. Study Population

**Figure 1:**
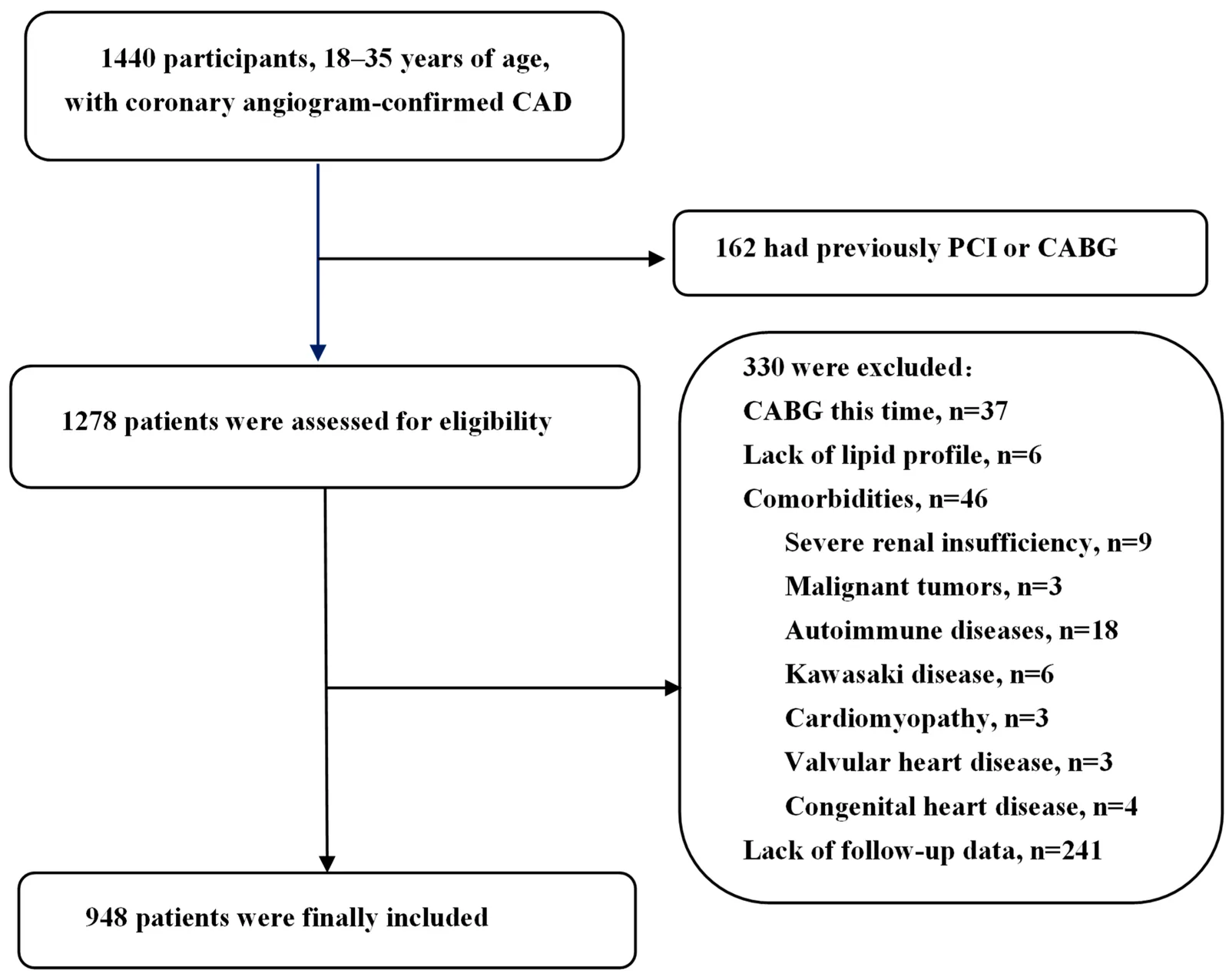
The flow chart of study population selection. CAD, coronary artery disease; PCI, percutaneous coronary intervention; CABG, coronary artery bypass grafting.

This single-center observational study included young patients aged 18–35 years who presented with chest tightness or chest pain and underwent coronary angiography at Beijing Anzhen Hospital between June 2013 and December 2021. Patients were eligible if coronary angiography confirmed a diagnosis of CAD during hospitalization. Those with a history of prior percutaneous coronary intervention or coronary artery bypass grafting (CABG), or who underwent CABG during the current hospitalization, were excluded. Other exclusion criteria included cardiomyopathy, congenital heart disease, valvular heart disease, Kawasaki disease, autoimmune diseases, severe hepatic or renal dysfunction (an estimated glomerular filtration rate [eGFR] < 30 mL/min/1.73 m^2^), active or previous malignancy, lack of lipid profile, and lack of follow-up data. After applying these criteria, a total of 948 patients (95.6% male) were enrolled (Figure 1). This study was conducted under the Declaration of Helsinki and was approved by the Institutional Ethics Committee of Beijing Anzhen Hospital (Ethics Approval No. 2026253x). Since the present study was retrospective, the informed consent was waived by the committee. 

### 2.2. Data Collection and Follow-Up

All baseline patient data analyzed in this study, including demographics, past medical history and pre-admission medications, laboratory findings, echocardiographic results, and coronary angiography data, were obtained from electronic medical records. During the index hospitalization, venous blood samples were collected after an overnight fast (≥12 h). The first fasting sample obtained after admission, generally on the morning after admission, was used to measure creatinine, eGFR, fasting blood glucose, triglycerides (TG), total cholesterol, HDL-C, LDL-C, uric acid, hemoglobin A1c, and high-sensitivity C-reactive protein (hs-CRP). Following admission, all patients underwent coronary angiography. To guarantee the objectivity and accuracy of the angiographic findings, two experienced clinicians jointly assessed and documented coronary artery stenosis. The severity of coronary lesions was then graded using the Gensini Score, with the total score calculated by summing individual lesion scores, each of which equals the product of a location-specific weighting factor and the degree of stenosis [13]. On the basis of coronary angiography, CAD was defined as ≥50% stenosis in at least one major epicardial coronary artery (diameter > 2 mm). ACS was diagnosed in accordance with the 2023 European Society of Cardiology guidelines [14], incorporating clinical presentation, electrocardiographic changes, and biochemical evidence of myocardial injury. In addition, body mass index (BMI) was obtained as the ratio of weight in kilograms to the square of height in meters. Hyperlipidemia was defined as total cholesterol ≥ 5.2 mmol/L or/and LDL-C ≥ 3.4 mmol/L. Familial hypercholesterolemia was established when patients met at least two of these criteria: LDL-C ≥ 4.68 mmol/L, tendon/skin xanthomas, or a personal history of familial hypercholesterolemia or premature CAD in the family [15].

Follow-up information was obtained by reviewing electronic health records after discharge. When this was not possible, telephone interviews were conducted with the patients or their direct relatives to confirm endpoint events, with follow-up continuing until July 2025. The primary endpoint was MACE, defined as a composite of all-cause mortality, non-fatal myocardial infarction (MI), non-fatal stroke, ischemia-driven revascularization, and hospitalization for heart failure. When multiple adverse events occurred, precedence was given to all-cause mortality, then to non-fatal stroke and MI, and finally to ischemia-driven revascularization. Only the first qualifying event was included in the analysis.

### 2.3. AIP Calculation and Grouping

AIP was calculated from the logarithmic transformation of the triglyceride (mmol/L) to HDL-C (mmol/L) ratio, expressed as log (TG/HDL-C) [16]. Patients were divided according to their Gensini Score into a low-score group (≤40) and a high-score group (>40). This cut-off was chosen because it corresponded to the upper range of the score distribution in our cohort (median: 34; IQR: 20–60) and represented a clinically meaningful threshold for distinguishing moderate from more severe coronary lesions in this young population. Patients were also stratified by the occurrence of MACE (non-MACE vs. MACE) and by AIP value (lower AIP group, <0.4; higher AIP group, ≥0.4). The AIP cut-off of 0.4 was used as an exploratory threshold because the restricted cubic spline analysis using a Cox regression model to examine the association between AIP and MACE showed a possible change in risk around this value.

### 2.4. Statistical Analysis

Data examination was performed using SPSS 26.0 (IBM SPSS Inc., Chicago, IL, USA), GraphPad Prism 5 (GraphPad Software, Inc., San Diego, CA, USA), and R software (version 4.3.3; R Foundation for Statistical Computing, Vienna, Austria). All statistical tests were two-tailed and *p* < 0.05 was considered statistically significant. The Kolmogorov–Smirnov test was used to assess normality. Based on the distribution pattern, continuous data are presented as either mean with standard deviation or median with interquartile range. For comparisons between groups, normally distributed continuous variables were analyzed using the independent samples t-test, while the Mann–Whitney U test was applied to non-normally distributed ones. Categorical data are expressed as counts and percentages, and group differences were evaluated with the chi-square test or Fisher’s exact test, as appropriate. Logistic regression analyses were performed to examine the association between AIP, treated as a continuous variable, and greater coronary lesion severity.

In the multivariable analysis, we adjusted for potential confounders, mainly including variables that differed significantly at baseline as well as established traditional risk factors. Specifically, sequential models were fitted as follows: Model 1 was unadjusted; Model 2 included adjustments for age and sex; Model 3 further accounted for drinking status, smoking, BMI, diabetes mellitus, hypertension, LDL-C, familial hypercholesterolemia, and pre-admission use of antiplatelet drugs, antihypertensive drugs, hypoglycemic drugs, and lipid-lowering drugs; Model 4 additionally adjusted for hs-CRP.

Kaplan–Meier curves were generated to illustrate the cumulative event rates across different AIP levels, and the log-rank test was used to compare the survival curves between groups. Univariable and multivariable Cox proportional hazards analyses were performed to evaluate the predictive value of AIP for MACE. AIP was assessed both as a nominal (categorical) and as a continuous variable. Multivariable Cox regression models were constructed sequentially, incorporating covariates with a univariate *p* value < 0.1, as follows: Model 1 included adjustments for smoking, diabetes mellitus, hyperlipidemia, and familial hypercholesterolemia; Model 2 further adjusted for pre-admission use of antiplatelet drugs, hypoglycemic drugs, and lipid-lowering drugs; Model 3 additionally adjusted for Gensini score, multivessel disease, and left ventricular ejection fraction (LVEF). To assess the consistency of the association between AIP and MACE across different populations, we performed subgroup analyses stratified by sex, smoking status, hypertension, diabetes mellitus, hyperlipidemia, and BMI (categorized as ≤28 and >28 kg/m^2^). In addition, restricted cubic spline (RCS) regression was performed to examine the non-linear relationships of AIP with greater coronary lesion severity (using Model 4) and with MACE (using Model 3).

## 3. Results

### 3.1. Baseline Characteristics of CAD Patients with Different Gensini Scores

The baseline clinical characteristics of the study population, stratified by Gensini score-defined severity of coronary stenosis, are detailed in Table 1. Of the 948 patients, 374 were classified into the high-score group and 574 into the low-score group. The high-score group demonstrated a significantly lower prevalence of alcohol consumption (*p* < 0.05). Although the high-score group exhibited a greater prevalence of smoking, diabetes, and familial hypercholesterolemia, these trends did not achieve statistical significance. Regarding pre-admission pharmacological therapy, utilization of antiplatelet, hypoglycemic, and lipid-lowering agents was significantly higher in the high-score group (all *p* < 0.05), but no such difference was seen for antihypertensive drugs. Biochemically, the high-score group was associated with markedly elevated levels of total cholesterol, LDL-C, AIP, and hs-CRP, alongside reduced HDL-C levels (all *p* < 0.05). In contrast, there were no discernible differences between the two groups with respect to age, sex, BMI, hypertension, family history of CAD, eGFR, HbA1c, or uric acid (all *p* > 0.05).

### 3.2. Relationship Between AIP and Greater Coronary Lesion Severity

**Figure 2:**
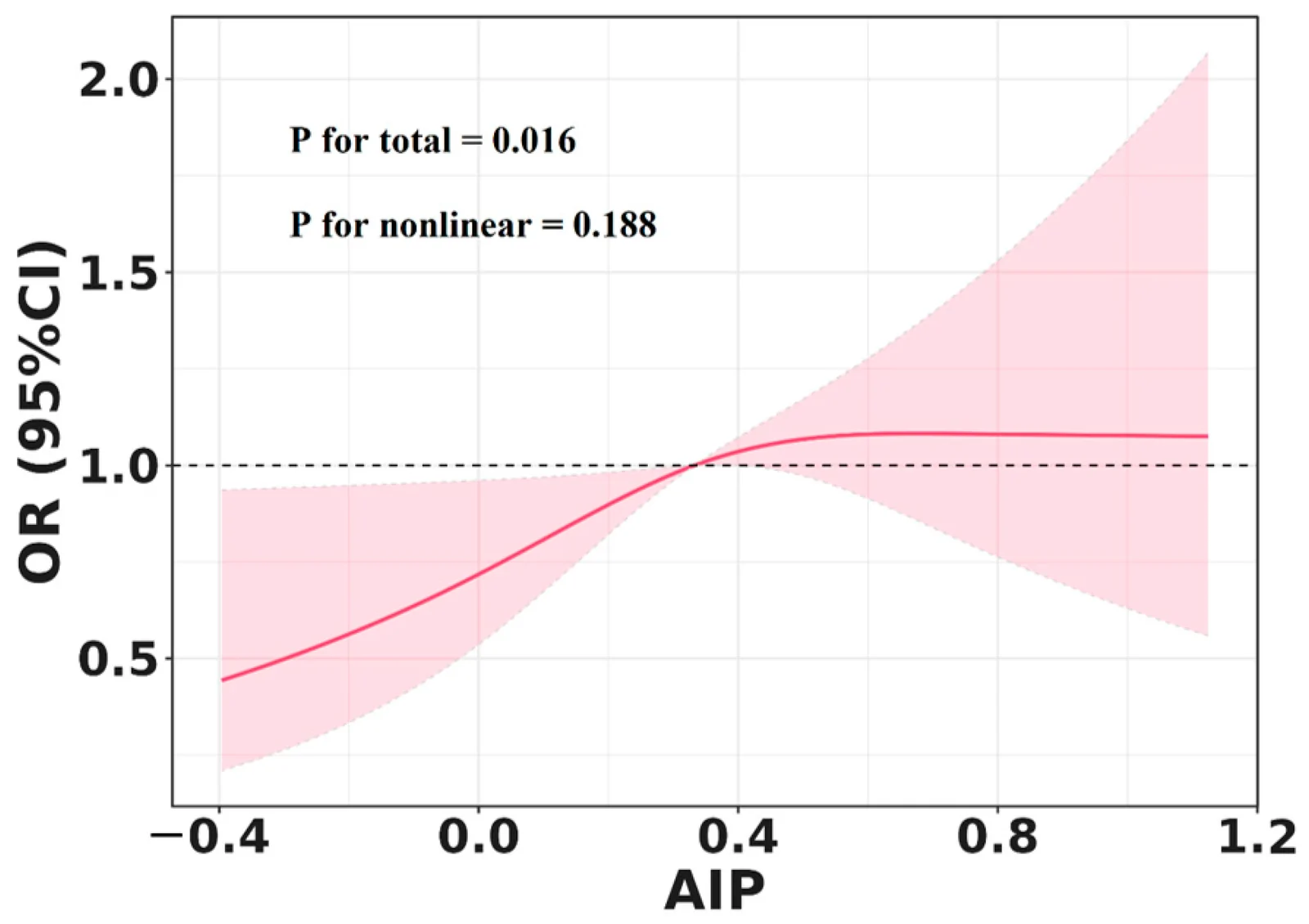
The relationship between AIP and greater coronary lesion severity with the RCS function. The logistic regression was adjusted for age, sex, smoking, BMI, diabetes mellitus, hypertension, LDL-C, familial hypercholesterolemia, antiplatelet drugs, antihypertensive drugs, hypoglycemic drugs, antilipidemic drugs and hs CRP. AIP, atherogenic index of plasma; BMI, body mass index; LDL-C, low-density lipoprotein cholesterol; hs CRP, high-sensitivity C-reactive protein; OR, odds ratio; CI, confidence interval.

Table 2 summarizes the multivariable logistic regression results for the association between AIP and greater coronary lesion severity. After full adjustment for drinking status, smoking, BMI, diabetes, hypertension, LDL-C, familial hypercholesterolemia, pre-admission use of antiplatelet, antihypertensive, hypoglycemic, and lipid-lowering drugs, as well as hs-CRP, AIP was also independently associated with severe coronary lesions (OR = 1.761; 95% CI: 1.052–2.948; *p* = 0.031). Based on Model 4, RCS regression was further performed to explore this relationship. The nonlinear component was not statistically significant (*p* for nonlinear = 0.188; Figure 2), yet a visual inspection indicated that the prevalence of severe coronary lesions generally rose with increasing AIP levels (*p* for overall = 0.016).

### 3.3. Baseline Characteristics of Patients Stratified by MACE and AIP

Table 3 compares the baseline clinical characteristics between patients with and without MACE. During a median follow-up of 60 months (IQR, 43–96), MACE occurred in 287 of the 948 enrolled patients, including 5 deaths, 64 nonfatal myocardial infarctions, 12 nonfatal strokes, 198 ischemia-driven revascularizations, and 8 hospitalizations for heart failure. Compared with the non-MACE group, patients with MACE had significantly higher Gensini scores, a greater prevalence of multivessel disease, and lower LVEF (all *p* < 0.05). They also showed significantly higher rates of smoking, hyperlipidemia, and familial hypercholesterolemia, as well as more frequent pre-admission antiplatelet use (all *p* < 0.05). Laboratory findings further revealed that the MACE group had significantly elevated levels of triglycerides, total cholesterol, LDL-C, AIP, and hs-CRP (all *p* < 0.05).

Similarly, Table 4 presents the baseline characteristics of patients stratified by AIP level. Compared with the lower AIP group (<0.4), the higher AIP group (≥0.4) had a greater proportion of males, a significantly higher prevalence of ACS, and higher Gensini scores (all *p* < 0.05). Regarding cardiovascular risk factors, the higher AIP group exhibited significantly higher proportions of smokers, higher BMI, and more frequent histories of hypertension, diabetes mellitus, and hyperlipidemia (all *p* < 0.05). In terms of pre-admission medications, antihypertensive and hypoglycemic drugs were used significantly more often in the higher AIP group (both *p* < 0.05). Laboratory data showed that the higher AIP group had significantly elevated levels of fasting blood glucose, HbA1c, triglycerides, total cholesterol, uric acid, and hs-CRP, along with lower HDL-C levels (all *p* < 0.05).

### 3.4. AIP and Cardiovascular Outcomes

**Figure 3:**
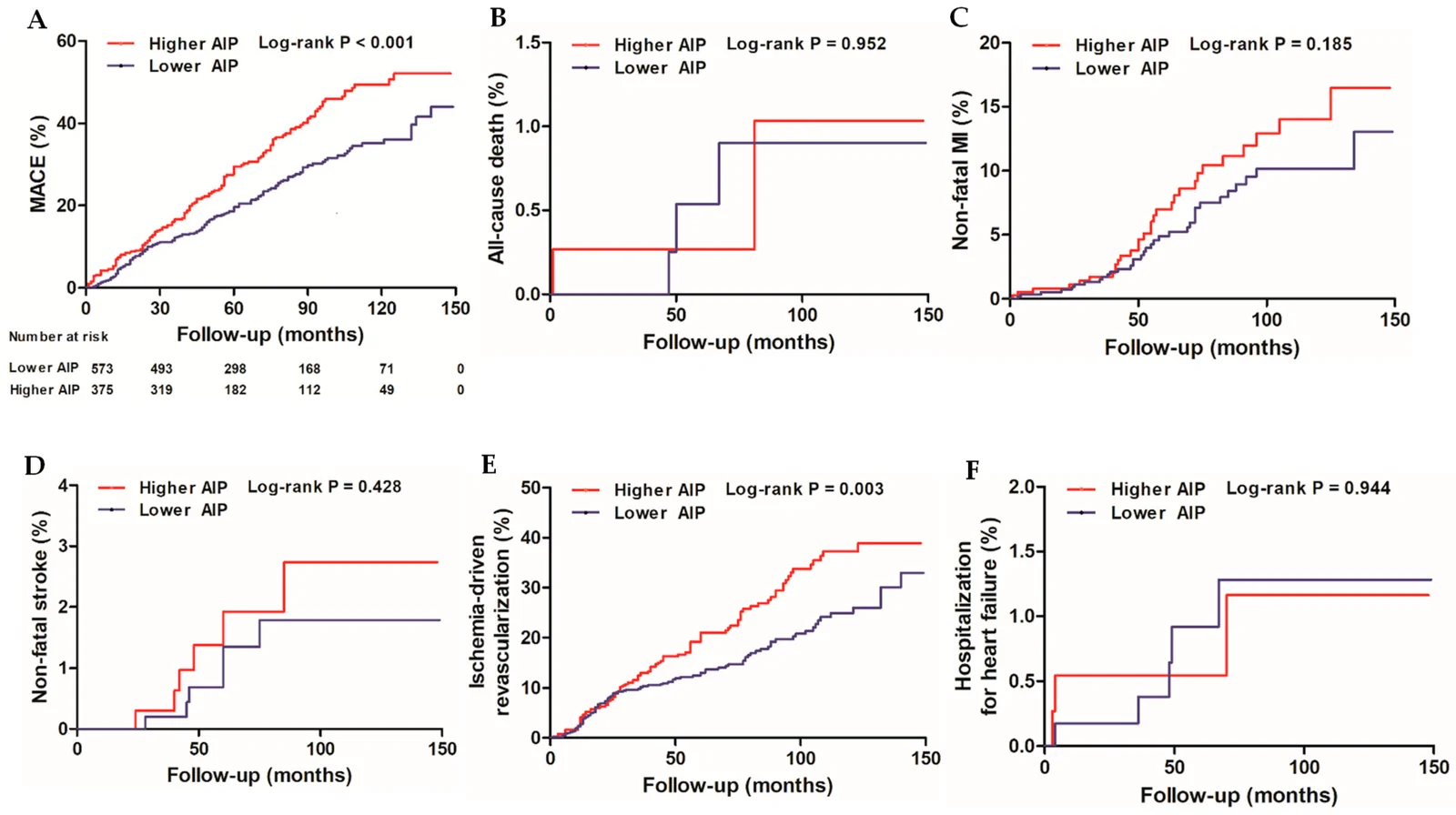
Kaplan–Meier survival curves between two AIP groups. (**A**), Kaplan–Meier survival curve of MACE; (**B**), Kaplan–Meier survival curve of all-cause death; (**C**), Kaplan–Meier survival curve of non-fatal MI; (**D**), Kaplan–Meier survival curve of non-fatal stroke; (**E**), Kaplan–Meier survival curve of ischemia-driven revascularization; (**F**), Kaplan–Meier survival curve of hospitalization for heart failure. AIP, atherogenic index of plasma; MACE major adverse cardiovascular events, MI myocardial infarction.

During follow-up, the higher AIP group had significantly higher rates of MACE and ischemia-driven revascularization compared with the lower AIP group (both *p* < 0.001; Table 5). These findings were supported by Kaplan–Meier estimates, which demonstrated greater cumulative incidences of both MACE and ischemia-driven revascularization in the higher AIP group (log-rank *p* < 0.001 for both; Figure 3A,E). In contrast, no significant differences were observed between the two groups in the rates or cumulative incidences of all-cause death, non-fatal MI, nonfatal stroke, or hospitalization for heart failure (all *p* > 0.05; Table 5 and Figure 3B–D,F).

**Figure 4:**
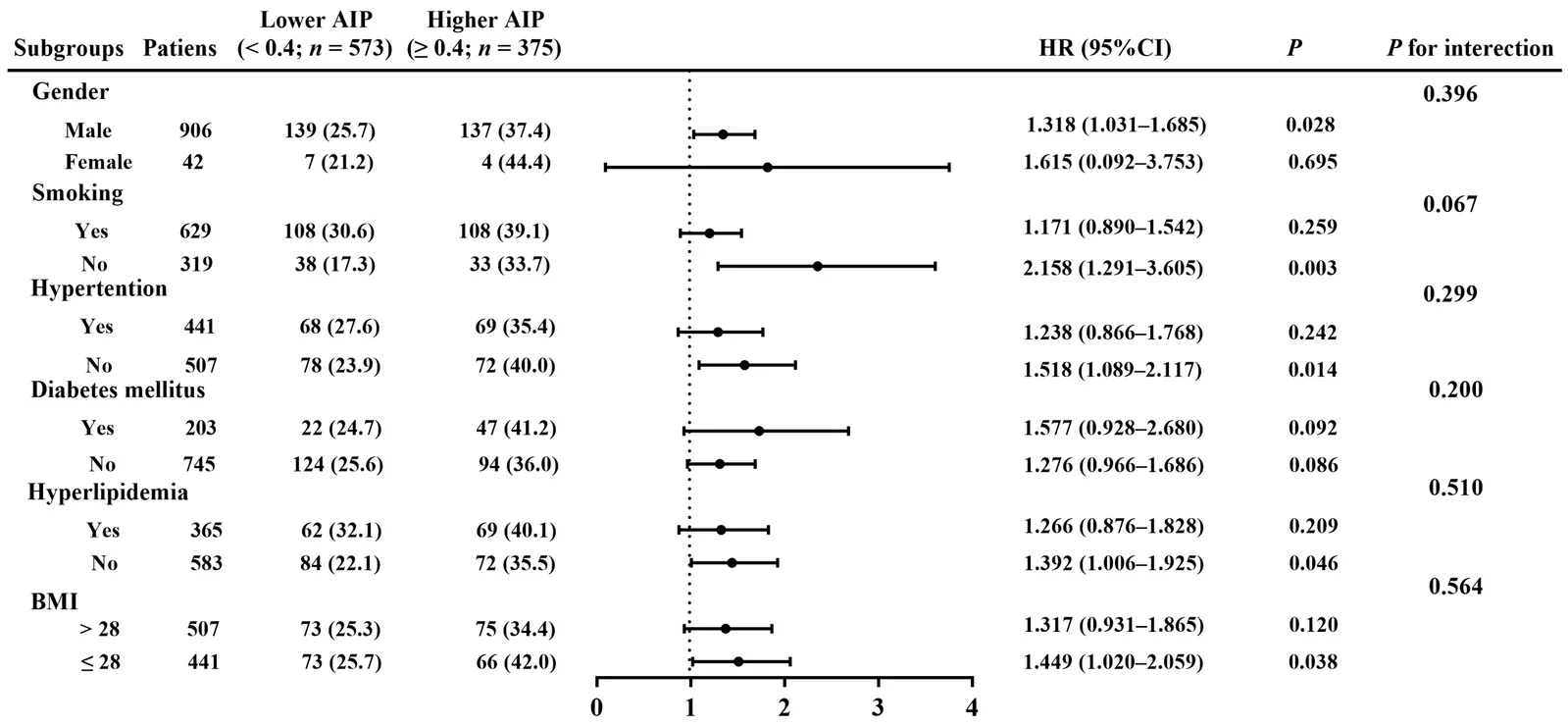
Subgroup analysis for the impact of AIP on the risk of MACE. AIP, atherogenic index of plasma; MACE major adverse cardiovascular events; BMI, body mass index; HR, hazard ratio; CI confidence interval.

Univariable Cox regression analyses identified smoking, hyperlipidemia, familial hypercholesterolemia, Gensini score, LVEF, multivessel disease, antiplatelet use, lipid-lowering therapy, and AIP (both as continuous and categorical variables) as independent influencing factors for MACE (all *p* < 0.05; Table 6). In sequential multivariable models, AIP as a categorical variable remained independently associated with MACE across all models, with the fully adjusted Model 3 yielding an HR of 1.350 (95% CI: 1.060–1.721, *p* = 0.015). However, when AIP was treated as a continuous variable, it did not retain statistical significance in Model 3 (*p* = 0.167) (Table 7). Further analysis of individual MACE components in the fully adjusted model revealed that AIP as a categorical variable remained a significant influencing factor for ischemia-driven revascularization (*p* = 0.014), whereas no significant associations were found for all-cause death, non-fatal MI, non-fatal stroke, or hospitalization for heart failure, irrespective of whether AIP was modeled as continuous or categorical (all *p* > 0.05) (Table 8). Subgroup analyses showed that the association between AIP (≥0.4 vs. <0.4) and MACE was generally consistent across all examined strata, with no significant interactions (all *p* for interaction > 0.05), although statistically significant associations were observed in specific subgroups including males, non-smokers, patients without hyperlipidemia or hypertension, and those with BMI ≤ 28 kg/m^2^ (Figure 4). 

**Figure 5:**
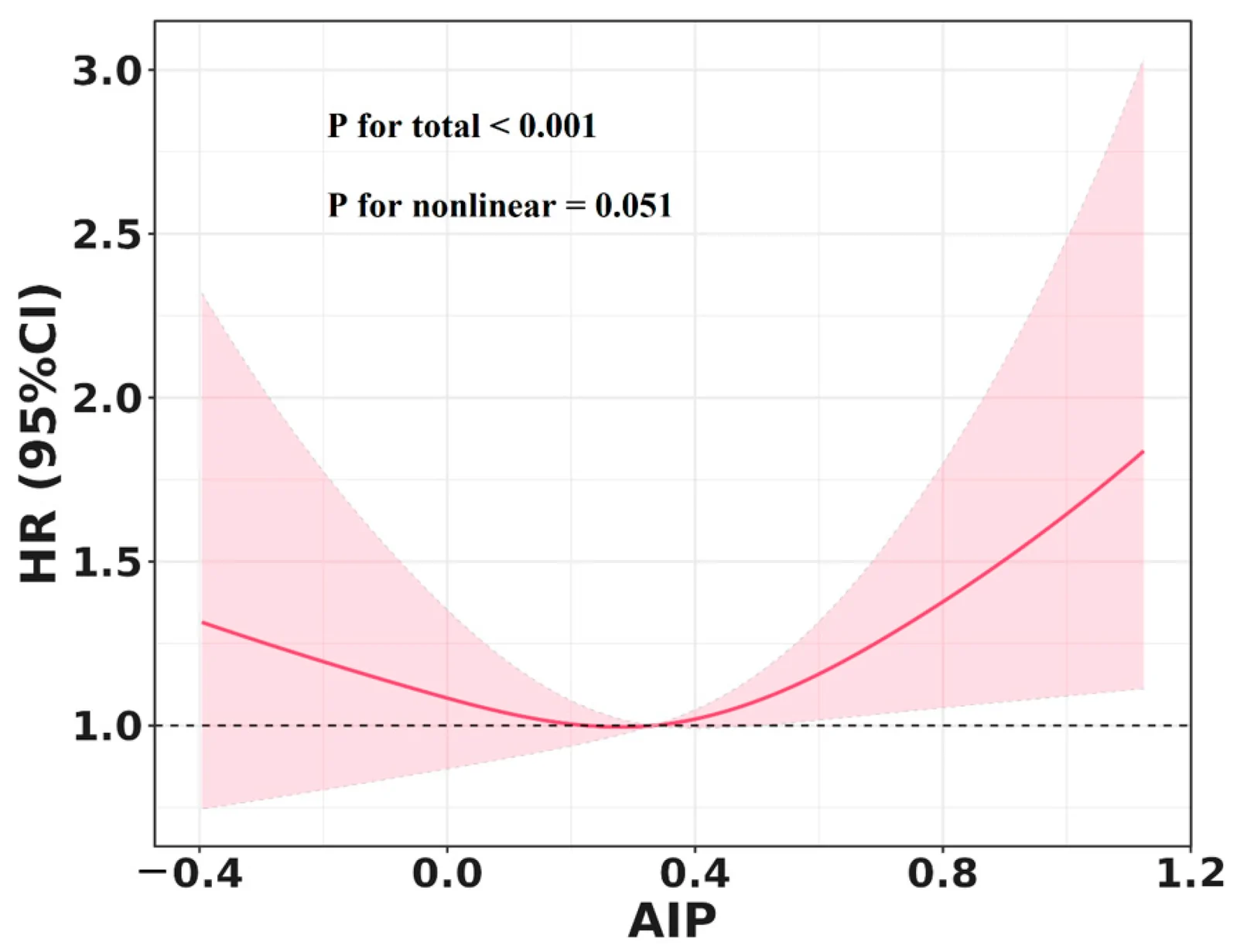
The relationship between AIP and MACE with the RCS function. The Cox regression analysis was adjusted for Model 3. RCS, restricted cubic splines; AIP, atherogenic index of plasma; MACE major adverse cardiovascular events, HR, hazard ratio; CI confidence interval.

Figure 5 illustrates the dose–response relationship between AIP and MACE using RCS regression, with adjustment for Model 3 covariates. The RCS curve suggested a possible U-shaped pattern, with a visual inflection point at approximately 0.4. For AIP levels below 0.4, the curve was relatively flat with a slight negative slope, suggesting a modest inverse trend. In contrast, for AIP values at or above 0.4, the slope became noticeably steeper and positive, indicating an increase in the risk of MACE with rising AIP. The overall association was highly significant (*p* for overall < 0.001), while the test for nonlinearity approached significance (*p* for nonlinear = 0.051), suggesting a borderline nonlinear relationship. These findings support the choice of 0.4 as a clinically meaningful threshold for stratifying patients by AIP in this cohort.

## 4. Discussion

This study identified AIP as an independent influencing factor for greater coronary lesion severity in young CAD patients, with a visual trend indicating that higher AIP levels were positively associated with more severe coronary lesions. Notably, when dichotomized at 0.4, categorical AIP was independently associated with MACE and ischemia-driven revascularization, whereas continuous AIP did not retain significance. The U-shaped dose–response curve, with a visual inflection at approximately 0.4, revealed a marked rise in risk above this cutoff, reinforcing its clinical value for risk stratification.

In recent years, the incidence of CAD among young populations has risen steadily, and CAD has become one of the leading contributors to sudden cardiac death in adults aged <35 years [17]. Although patients ≤35 years account for only approximately 1% of all CAD cases, they are at a higher risk of repeat revascularization during a mean follow-up of 5 years and have a higher rate of recurrent myocardial infarction than those aged 36–40 years [18]. This substantial disease burden not only compromises patients’ quality of life and long-term health but also places considerable economic and care-related strain on families and society. Therefore, a deeper understanding of both traditional and emerging risk predictors is essential for preventing adverse cardiovascular events in young patients with CAD.

As a practical composite lipid parameter that integrates the balance between atherogenic and protective lipoproteins, AIP may influence the progression of coronary atherosclerosis via multiple mechanisms. Dobiášová et al. first reported that AIP is strongly and negatively correlated with LDL particle diameter (r = −0.776), and that elevated AIP reliably reflects an increased proportion of small dense low-density lipoprotein (sdLDL) [16]. Owing to its smaller size, sdLDL penetrates the vascular endothelium more readily and is more susceptible to oxidation; the resulting oxidized LDL is subsequently engulfed by macrophages and converted into foam cells, a critical early event in atherosclerotic plaque formation [6]. Furthermore, sdLDL promotes atherosclerosis by upregulating endothelial adhesion molecules and activating oxygen radicals, thereby facilitating lipid peroxidation and other pro-atherogenic processes [19,20]. Importantly, an elevated AIP is not simply a marker of higher sdLDL—it also reflects increased TG, which can directly damage the endothelium and amplify inflammatory responses [21], alongside reduced HDL-C, which compromises the protective functions of reverse cholesterol transport, antioxidant defense, and anti-inflammatory activity [22]. Collectively, AIP encapsulates three interrelated lipid abnormalities—elevated TG, decreased HDL-C, and an increased sdLDL fraction—and thereby serves as a comprehensive indicator that systematically drives the initiation and progression of atherosclerosis.

Previous studies from our center on young (≤35 years of age) patients with ACS demonstrated that AIP levels increased significantly with rising Gensini scores and the number of diseased vessels. Moreover, when patients were stratified by AIP quartiles, the prevalence of ACS, AMI, and unstable angina, as well as Gensini scores, all increased progressively with higher AIP levels [23]. Similarly, Song et al., in a study of 163 patients with premature CAD (defined as age <55 years in men and <65 years in women), found that AIP levels rose significantly with increasing severity of coronary lesions (stratified by Gensini score), and AIP was significantly positively correlated with the Gensini score (*r* = 0.573, *p* < 0.001) [24]. In another study of 423 CAD patients aged 18–59 years, patients in the high Gensini score group (>median 54.91) had significantly higher AIP levels than those in the low score group. Among all lipid parameters, AIP showed the strongest positive correlation with the Gensini score (r = 0.558, *p* < 0.001). After adjustment for confounders, AIP remained an independent predictor of severe coronary lesions (OR = 1.575, *p* < 0.001) [25]. Collectively, these findings from different centers and across various age groups consistently demonstrate that AIP, as a simple and readily available composite lipid index, effectively reflects coronary atherosclerotic burden and holds considerable value in evaluating the severity of coronary lesions in young populations. In line with these observations, our present study yielded similar results: higher AIP levels were associated with a higher prevalence of ACS, elevated Gensini scores, and a trend toward decreased LVEF, reflecting a more severe disease phenotype and greater cardiac functional impairment. Furthermore, defining severe coronary artery disease as a Gensini score >40, multivariate logistic regression analysis showed that AIP was independently associated with severe coronary lesions. These findings highlight that AIP not only facilitates early identification of severe coronary lesions in young individuals but also serves as a convenient tool for clinical risk stratification, providing a valuable reference for guiding intensive lipid-lowering therapy and individualized intervention strategies in high-risk patients.

Regarding the prognostic value of AIP, previous studies have demonstrated that AIP independently predicts the incidence of cardiovascular disease (CVD) and related mortality [26], and may outperform individual cholesterol parameters as a more reliable predictor of death [27]. Nonetheless, evidence specifically targeting younger populations remains scarce. A study using data from the National Health and Nutrition Examination Survey (NHANES) spanning 2005–2018, with a median follow-up of 95 months, reported a J-shaped association between AIP and all-cause mortality. In the 40–60 years age subgroup, AIP showed a significant positive association with both all-cause and CVD mortality, suggesting its potential utility as a prognostic indicator of future mortality risk in this age group [28]. Similarly, Ran et al. [5] conducted a retrospective cohort study of 721 patients with premature CAD (defined as age <45 years in men and <55 years in women), with a median follow-up of 52 months, and found that the highest AIP tertile (>0.255) was associated with the greatest incidence of MACE. This association remained significant even after adjusting for traditional risk factors and other confounders, indicating that AIP independently predicts MACE in this population. In our present study, when AIP was treated as a continuous variable, no significant association was observed with MACE or its component events. However, when AIP was dichotomized using a threshold of ≥0.4, it was independently associated with both MACE and ischemia-driven revascularization. RCS analysis revealed a possible U-shaped relationship between AIP and MACE, suggesting that these seemingly conflicting findings may be attributable to this nonlinearity. Such U-shaped associations between AIP and various cardiovascular outcomes have also been reported in prior studies [29,30], and the underlying biology may involve distinct pathways at the two extremes of the AIP distribution. At the upper end, elevated AIP typically reflects an excess of triglyceride-rich remnant lipoproteins, and elevated remnant cholesterol has been shown to significantly increase the risk of composite cardiac events in patients with premature CAD [31]. At the lower end, very low AIP levels may stem from markedly reduced triglycerides or abnormally elevated HDL-C. The latter can be partly explained by loss-of-function variants in the *SCARB1* gene, which, despite raising HDL-C levels, paradoxically increase coronary artery disease risk by compromising HDL functionality [32]. This suggests that the lower segment of the AIP spectrum does not signify a genuine lipid-protective effect, but rather reflects a discordance between HDL quantity and function. Collectively, these findings indicate that using AIP as a categorical variable may more effectively identify high-risk individuals at both extremes of the U-shaped curve, thereby circumventing the attenuation of effect estimates resulting from nonlinearity when AIP is modeled continuously, and highlighting that threshold-based assessment may enhance risk stratification in clinical practice.

Previous investigations into the relationship between AIP and MACE across various subgroups have produced inconsistent findings, likely due to differences in study populations, racial heterogeneity, and variations in the definitions of cardiovascular outcomes. For instance, Wang et al. reported a stronger link in male ACS patients [33], whereas Ran et al. observed a more pronounced association in female patients with premature CAD (defined as age <45 years in men and <55 years in women) [5]. Consistent with the former, our study identified a significant association in males; in females, although the HR exceeded 1, it did not reach statistical significance, probably owing to the limited female sample size and consequent insufficient statistical power. With regard to BMI stratification, Kan et al. demonstrated that AIP’s prognostic value for MACE was confined to ACS patients with BMI < 24 kg/m^2^ [10], and a separate study similarly reported a significant association between AIP and cardiovascular death among individuals with BMI < 25 kg/m^2^ [28]. Our findings further showed that the predictive effect of AIP was stronger in participants with BMI ≤ 28 kg/m^2^ than in obese individuals, implying that in populations with established metabolic disturbances, the lipid-related risk pathway captured by AIP may be overshadowed or attenuated by other more dominant pathogenic processes. Likewise, among subgroups defined by other conventional risk factors, including smoking, diabetes, hyperlipidemia, and hypertension, the HRs for AIP were consistently above 1 but failed to achieve statistical significance. This may reflect competing effects from these traditional risk factors, which dilute the independent predictive capacity of AIP. Notably, although the association between AIP and MACE reached statistical significance in some subgroups without conventional risk factors, no significant interactions were observed. Thus, it remains a hypothesis that AIP might more purely reflect future cardiovascular risk through lipid metabolic derangements in such patients, and this requires further validation.

Several limitations of this study should be acknowledged. First, as a single-center retrospective study, selection and information biases may exist, and the generalizability of our findings to other regions or populations requires further validation. Second, the sex distribution was markedly imbalanced, with female patients accounting for only 4.4% of the cohort (Supplementary Table S1). Although this is consistent with the known sex-specific epidemiology of young CAD, the limited female sample size inevitably undermined the statistical power for subgroup analyses and compromised the reliability of sex-specific conclusions. Third, the small number of events for certain MACE components, such as all-cause death, non-fatal stroke and hospitalization for heart failure, precluded robust analyses of these individual endpoints. Fourth, AIP was calculated from a single fasting blood sample obtained during the index hospitalization, generally on the morning after admission, rather than before the acute event or after stabilization. Because this single measurement did not account for longitudinal changes or fluctuations during follow-up, the influence of temporal variability in AIP on long-term prognosis may have been underestimated. In addition, among ACS patients, acute-phase stress, inflammation, and in-hospital treatments may have affected lipid levels. Therefore, the temporal relationship between AIP and outcomes should be interpreted cautiously. Fifth, the optimal AIP cutoff of ≥ 0.4 was derived from the RCS curve within this specific cohort, and its applicability to other populations or ethnicities remains to be established. Lastly, detailed information on lipid-lowering agents and novel hypoglycemic drugs, including their specific types and dosages, was not available, and these medications may have potentially influenced lipid profiles and clinical outcomes. These limitations should be carefully considered when interpreting our results.

## 5. Conclusions

In conclusion, AIP exhibited a positive correlation with greater coronary lesion severity and MACE in young CAD patients (≤35 years of age). When dichotomized at a threshold of 0.4, AIP demonstrates robust prognostic value for MACE, primarily driven by ischemia-driven revascularization, whereas continuous AIP fails to retain independent significance, possibly reflecting a borderline U-shaped trend. Given its simplicity, low cost, and wide availability, AIP could be readily integrated into routine clinical practice to facilitate early identification of high-risk young CAD patients, guide timely intensive lipid-lowering interventions, and inform individualized management strategies. However, large-scale, multicenter, prospective studies are warranted to externally validate the optimal threshold and to further elucidate the underlying mechanisms linking AIP to adverse outcomes in this unique population.

## Figures and Tables

**Table 1:** Basic clinical characteristics of CAD patients with different Gensini scores.

Characteristics	High-Score Group (*n* = 374)	Low-Score Group (*n* = 574)	*p*
General characteristics			
Age (years)	33 (31–34)	33 (31–34)	0.856
Male, *n* (%)	360 (96.3)	546 (95.1)	0.407
Drinker, *n* (%)	58 (15.5)	123 (21.5)	0.023
Medical history and coronary risk factors			
Smoking, *n* (%)	262 (70.1)	367 (64.0)	0.056
BMI (kg/m^2^)	28.40 (25.86–31.20)	28.08 (25.56–31.10)	0.339
Hypertension, *n* (%)	177 (47.3)	264 (46.0)	0.688
Diabetes mellitus, *n* (%)	92 (24.6)	111 (19.3)	0.054
Family history of CAD, *n* (%)	37 (9.9)	55 (9.6)	0.874
Familial hypercholesterolemia, *n* (%)	14 (3.7)	10 (1.7)	0.055
Pre-admission Medications			
Antiplatelet drugs, *n* (%)	14 (3.7)	9 (1.6)	0.033
Antihypertensive drugs, *n* (%)	82 (21.9)	109 (19.0)	0.271
Hypoglycemic drugs, *n* (%)	41 (11.0)	35 (6.1)	0.007
Antilipidemic drugs, *n* (%)	28 (7.5)	14 (2.4)	<0.001
Laboratory results			
eGFR (mL/min/1.73 m^2^)	116.67 (103.90–122.41)	115.93 (105.79–122.19)	0.709
Hemoglobin A1c (%)	5.7 (5.4–6.8)	5.6 (5.3–6.2)	0.104
Triglyceride (mmol/L)	1.93 (1.42–2.93)	1.86 (1.27–2.72)	0.067
Total cholesterol (mmol/L)	4.70 (3.73–5.66)	4.43 (3.54–5.37)	0.006
HDL-C (mmol/L)	0.87 (0.75–1.01)	0.90 (0.79–1.03)	0.016
LDL-C (mmol/L)	2.86 (2.14–3.81)	2.68 (2.01–3.45)	0.007
Uric acid (µmol/L)	411.15 (348.48–470.93)	409.30 (351.20–469.70)	0.689
AIP	0.36 (0.20–0.57)	0.31 (0.12–0.51)	0.011
hs CRP (mg/L)	3.55 (1.00–11.21)	2.55 (0.97–7.84)	0.028

Notes: Data are expressed as medians with interquartile range for continuous variables with non-normal distribution and number (%) for categorical variables. Bold values indicate statistical significance. CAD, coronary artery disease; BMI, body mass index; eGFR, estimated glomerular filtration rate; HDL-C, high-density lipoprotein cholesterol; LDL-C, low-density lipoprotein cholesterol; AIP, atherogenic index of plasma; hs CRP, high-sensitivity C-reactive protein.

**Table 2:** Univariable and multivariable logistic regression analysis for greater coronary lesion severity.

Variables		OR	95% Cl	*p*
AIP	Model 1	1.661	1.093–2.523	**0.017**
	Model 2	1.640	1.076–2.499	**0.021**
	Model 3	1.766	1.055–2.957	**0.030**
	Model 4	1.761	1.052–2.948	**0.031**

Notes: Model 1 was unadjusted univariate analysis; Model 2 was adjusted for age and sex; Model 3 was further adjusted for drinker, smoking, BMI, diabetes mellitus, hypertension, LDL-C, familial hypercholesterolemia, antiplatelet drugs, antihypertensive drugs, hypoglycemic drugs and antilipidemic drugs; Model 4 was further adjusted for hs CRP. Bold values indicate statistical significance. AIP, atherogenic index of plasma; BMI, body mass index; LDL-C, low-density lipoprotein cholesterol; hs CRP, high-sensitivity C-reactive protein; OR, odds ratio; CI, confidence interval.

**Table 3:** Basic clinical characteristics between MACE groups and non-MACE groups in patients with CAD.

Variables	MACE (*n* = 287)	Non-MACE (*n* = 661)	*p*
General characteristics			
Age (years)	33 (30–34)	33 (31–34)	0.350
Male, *n* (%)	276 (96.2)	630 (95.3)	0.549
Drinker, *n* (%)	49 (17.0)	132 (20.0)	0.282
LVEF	60 (55–65)	62 (56–66)	**0.017**
Gensini score	44 (24–76)	32 (20–52)	**<0.001**
Multivessel disease, *n* (%)	195 (67.9)	292 (44.2)	**<0.001**
Medical history and coronary risk factors			
Smoking, *n* (%)	216 (75.3)	413 (62.6)	**<0.001**
BMI (kg/m^2^)	27.92 (25.55–31.45)	28.21 (25.71–30.93)	0.940
Hypertension, *n* (%)	137 (47.7)	304 (46.0)	0.621
Diabetes mellitus, *n* (%)	69 (24.0)	134 (20.3)	0.194
Hyperlipidemia, *n* (%)	131 (45.6)	234 (35.4)	**0.003**
Family history of CAD, *n* (%)	27 (9.4)	65 (9.8)	0.839
Familial hypercholesterolemia, *n* (%)	13 (4.5)	11 (1.7)	**0.010**
Pre-admission Medications			
Antiplatelet drugs, *n* (%)	13 (4.5)	10 (1.5)	**0.006**
Antihypertensive drugs, *n* (%)	59 (20.6)	132 (20.0)	0.836
Hypoglycemic drugs, *n* (%)	27 (9.4)	49 (7.4)	0.299
Antilipidemic drugs, *n* (%)	18 (6.3)	24 (3.6)	0.069
Laboratory results			
Creatinine (µmol/L)	73.00 (66.24–83.45)	75.00 (66.60–83.85)	0.467
eGFR (mL/min/1.73 m^2^)	116.71 (105.97–124.39)	115.95 (105.00–121.49)	0.154
Fasting blood glucose (mmol/L)	5.35 (4.86–6.52)	5.37 (4.91–6.45)	0.852
Hemoglobin A1c (%)	5.7 (5.3–6.7)	5.6 (5.3–6.4)	0.500
Triglyceride (mmol/L)	2.11 (1.41–3.28)	1.84 (1.31–2.64)	**0.004**
Total cholesterol (mmol/L)	4.77 (3.74–5.85)	4.45 (3.57–5.39)	**0.005**
HDL-C (mmol/L)	0.87 (0.76–1.01)	0.90 (0.78–1.03)	0.057
LDL-C (mmol/L)	2.86 (2.16–3.82)	2.70 (2.04–3.49)	**0.009**
Uric acid (µmol/L)	421.20 (354.45–484.70)	405.40 (348.48–467.43)	0.116
AIP	0.39 (0.19–0.60)	0.31 (0.14–0.51)	**0.001**
hs CRP (mg/L)	3.72 (1.08–10.00)	2.63 (0.97–7.91)	**0.034**

Notes: Data are expressed as medians with interquartile range for continuous variables with non-normal distribution and number (%) for categorical variables. Bold values indicate statistical significance. MACE, major adverse cardiovascular events; CAD, coronary artery disease; LVEF, left ventricular ejection fraction; BMI, body mass index; eGFR, estimated glomerular filtration rate; HDL-C, high-density lipoprotein cholesterol; LDL-C, low-density lipoprotein cholesterol; AIP, atherogenic index of plasma; hs CRP, high-sensitivity C-reactive protein.

**Table 4:** Baseline characteristics of the study population in two groups of AIP.

Variables	Lower AIP (<0.4; *n* = 573)	Higher AIP (≥0.4; *n* = 375)	*p*
General characteristics			
Age (years)	33 (31–34)	33 (31–34)	0.271
Male, *n* (%)	540 (94.2)	366 (97.6)	**<0.015**
Drinker, *n* (%)	99 (17.3)	82 (21.9)	0.075
ACS	140 (24.4)	349 (93.1)	**<0.001**
LVEF	62 (56–66)	60 (55–65)	0.053
Gensini score	32 (20–58)	36 (20–64)	**0.035**
Medical history and coronary risk factors			
Smoking, *n* (%)	353 (61.6)	276 (73.8)	**<0.001**
BMI (kg/m^2^)	27.78 (25.25–30.47)	28.70 (26.37–31.93)	**<0.001**
Hypertension, *n* (%)	246 (42.9)	195 (52.0)	**0.006**
Diabetes mellitus, *n* (%)	89 (15.5)	114 (30.4)	**<0.001**
Hyperlipidemia, *n* (%)	193 (33.7)	172 (45.9)	**<0.001**
Family history of CAD, *n* (%)	51 (8.9)	41 (10.9)	0.301
Familial hypercholesterolemia, *n* (%)	18 (3.1)	6 (1.6)	0.140
Pre-admission Medications			
Antiplatelet drugs, *n* (%)	15 (2.6)	8 (2.1)	0.635
Antihypertensive drugs, *n* (%)	103 (18.0)	88 (23.5)	**0.039**
Hypoglycemic drugs, *n* (%)	30 (5.2)	46 (12.3)	**<0.001**
Antilipidemic drugs, *n* (%)	25 (4.4)	17 (4.5)	0.901
Laboratory results			
Creatinine (µmol/L)	73.90 (65.50–83.15)	75.20 (68.25–84.60)	0.082
eGFR (mL/min/1.73 m^2^)	115.91 (105.99–122.54)	116.49 (104.42–121.66)	0.460
Fasting blood glucose (mmol/L)	5.24 (4.83–5.84)	5.70 (5.03–7.72)	**<0.001**
Hemoglobin A1c (%)	5.5 (5.2–6.0)	5.8 (5.5–7.4)	**<0.001**
TG (mmol/L)	1.44 (1.10–1.84)	3.20 (2.55–4.35)	**<0.001**
Total cholesterol (mmol/L)	4.25 (3.44–5.27)	4.96 (4.15–5.80)	**<0.001**
HDL-C (mmol/L)	0.96 (0.85–1.09)	0.80 (0.70–0.91)	**<0.001**
LDL-C (mmol/L)	2.68 (1.98–3.62)	2.86 (2.15–3.56)	0.175
Uric acid (µmol/L)	393.60 (340.70–455.40)	430.40 (369.83–501.48)	**<0.001**
AIP	0.20 (0.04–0.31)	0.59 (0.50–0.73)	**<0.001**
hs CRP (mg/L)	2.38 (0.89–7.69)	4.00 (1.26–9.81)	**<0.001**

Notes: Data are expressed as medians with interquartile range for continuous variables with non-normal distribution and number (%) for categorical variables. Bold values indicate statistical significance. AIP, atherogenic index of plasma; ACS, acute coronary syndrome; LVEF, left ventricular ejection fraction; BMI, body mass index; CAD, coronary artery disease; eGFR, estimated glomerular filtration rate; HDL-C, high-density lipoprotein cholesterol; LDL-C, low-density lipoprotein cholesterol; hs CRP, high-sensitivity C-reactive protein.

**Table 5:** Incidence of MACE and each component in two groups of AIP.

Variables	Lower AIP (<0.4; *n* = 573)	Higher AIP (≥0.4; *n* = 375)	*p*
MACE, *n* (%)	146 (25.5)	141 (37.6)	**<0.001**
All-cause death, *n* (%)	3 (0.5)	2 (0.5)	1.000
Non-fatal MI, *n* (%)	34 (5.9)	30 (8.0)	0.215
Non-fatal stroke, *n* (%)	6 (1.0)	6 (1.6)	0.457
Ischemia-driven revascularization, *n* (%)	98 (17.1)	100 (26.7)	**<0.001**
Hospitalization for heart failure	5 (0.9)	3 (0.8)	1.000

Notes: Data are expressed as number (%) for categorical variables. Bold values indicate statistical significance. AIP, atherogenic index of plasma; MACE, major adverse cardiovascular events; MI, myocardial infarction.

**Table 6:** Univariable Cox proportional hazards analyses for MACE.

	As Continuous Variate ^a^			As Nominal Variate ^b^		
	HR	95% Cl	*p*	HR	95% Cl	*p*
Model 1	1.392	0.942–2.057	0.096	1.404	1.104–1.786	**0.006**
Model 2	1.460	0.988–2.156	0.057	1.415	1.112–1.802	**0.005**
Model 3	1.320	0.891–1.955	0.167	1.350	1.060–1.721	**0.015**

Notes: ^a^ The HR was evaluated by per 1-unit decrease of AIP; ^b^ The HR was evaluated regarding the lower group of AIP as reference. Bold values indicate statistical significance. MACE, major adverse cardiovascular events; AIP, atherogenic index of plasma; HR: hazard ratio; CI, confidence interval.

**Table 7:** Predictive value of AIP for MACE in different Cox proportional hazards models.

Variables	Univariate Analysis		
	HR	95% Cl	*p*
Age	0.991	0.952–1.032	0.675
Male	1.270	0.695–2.320	0.437
Smoking	1.617	1.236–2.114	**<0.001**
Hypertension	1.009	0.800–1.272	0.941
Diabetes mellitus	1.288	0.982–1.690	0.067
Hyperlipidemia	1.342	1.064–1.693	**0.013**
Familial hypercholesterolemia	1.914	1.097–3.342	**0.022**
LDL-C	1.006	0.989–1.023	0.507
Gensini score	1.012	1.009–1.015	**<0.001**
LVEF	0.982	0.968–0.996	**0.011**
Multivessel disease	2.326	1.815–2.983	**<0.001**
Antiplatelet drugs	1.940	1.112–3.384	**0.020**
Antihypertensive drugs	0.975	0.732–1.298	0.860
Hypoglycemic drugs	1.477	0.993–2.198	0.054
Antilipidemic drugs	1.797	1.114–2.899	**0.016**
AIP as a continuous variable ^a^	1.659	1.154–2.383	**0.006**
AIP as a nominal variable ^b^	1.517	1.204–1.913	**<0.001**
hs CRP	1.004	0.993–1.015	0.479

Notes: ^a^ The HR was evaluated by per 1-unit decrease of AIP; ^b^ The HR was evaluated regarding the lower group of AIP as reference. Model 1 was adjusted for smoking, diabetes mellitus, hyperlipidemia and familial hypercholesterolemia; Model 2 was further adjusted for antiplatelet drugs, hypoglycemic drugs and antilipidemic drugs; Model 3 was further adjusted for Gensini score, multivessel disease and LVEF. Bold values indicate statistical significance. MACE, major adverse cardiovascular events; AIP, atherogenic index of plasma; LVEF, left ventricular ejection fraction; HR: hazard ratio; CI, confidence interval.

**Table 8:** Predictive value of AIP for MACE and each component in univariate and multivariate Cox regression analysis.

	Univariate Analysis			Multivariate Analysis ^a^		
	HR	95% Cl	*p*	HR	95% Cl	*p*
AIP as a continuous variable ^b^						
MACE	1.659	1.154–2.383	**0.006**	1.320	0.891–1.955	0.167
All-cause death	0.144	0.008–2.236	0.191	0.067	0.004–1.226	0.068
Non-fatal MI	2.209	1.039–4.697	**0.039**	1.393	0.608–3.188	0.433
Non-fatal stroke	0.955	0.153–5.961	0.961	1.626	0.231–11.455	0.626
Ischemia-driven revascularization	1.793	1.162–2.767	**0.008**	1.467	0.916–2.349	0.111
Hospitalization for heart failure	0.172	0.017–1.729	0.135	0.124	0.011–1.399	0.091
AIP as a nominal variable ^c^						
MACE	1.517	1.204–1.913	**<0.001**	1.350	1.060–1.721	**0.015**
All-cause death	1.056	0.176–6.319	0.953	0.539	0.087–3.344	0.507
Non-fatal MI	1.391	0.852–2.273	0.187	1.107	0.660–1.856	0.699
Non-fatal stroke	1.573	0.507–4.879	0.432	2.038	0.598–6.949	0.255
Ischemia-driven revascularization	1.600	1.211–2.115	**0.001**	1.441	1.076–1.930	**0.014**
Hospitalization for heart failure	0.950	0.227–3.977	0.944	0.879	0.207–3.740	0.862

Notes: ^a^ The multivariate analysis was performed by using Model 3; ^b^ The HR was evaluated by per 1-unit decrease of AIP; ^c^ The HR was evaluated regarding the lower group of AIP as reference. MACE, major adverse cardiovascular events; AIP, atherogenic index of plasma; MI, myocardial infarction; HR: hazard ratio; CI, confidence interval. Bold values indicate statistical significance.

## Data Availability

The individual-level datasets generated and analyzed in the current study are not publicly available due to patient confidentiality and institutional data protection requirements. An anonymized aggregate dataset and analysis code may be made available from the corresponding author on reasonable request, subject to institutional approval.

## Supplementary Materials

The following supporting information can be downloaded at: https://www.mdpi.com/article/10.3390/jcm15208092/s1, Table S1: Baseline Characteristics of Female Patients with Coronary Artery Disease Aged ≤ 35 Years.

## Abbreviations

CAD, coronary artery disease; AIP, atherogenic index of plasma; MACE, major adverse cardiovascular events; HDL-C, high-density lipoprotein cholesterol; LDL-C, low-density lipoprotein cholesterol; TG, triglyceride; ACS, acute coronary syndrome; AMI, acute myocardial infarction; CABG, coronary artery bypass grafting; PCI, percutaneous coronary intervention; BMI, body mass index; eGFR, estimated glomerular filtration rate; hs-CRP, high-sensitivity C-reactive protein; LVEF, left ventricular ejection fraction; RCS, restricted cubic spline.
