# Supplementary material for: Prognostic Value of the Atherogenic Index of Plasma for Severity and Cardiovascular Outcomes in Young Patients (≤35 Years) with Coronary Artery Disease: A Retrospective Study
Source: J Clin Med. 2026 Oct 19;15(20):8092. doi: 10.3390/jcm15208092 (PMC13706528; doi:10.3390/jcm15208092)
Supplement: Supplementary file 1 [file jcm-15-08092-s001.zip › jcm-4531759-Supplementary.pdf]

Table S1. Baseline Characteristics of Female Patients with Coronary Artery Disease Aged ≤ 35 Years

Variables	Total (n = 948)	Female (n = 42)
General characteristics		
Age (years)	33 (31-34)	32 (30-34)
Drinker, n (%)	181 (19.1)	0 (0.0)
LVEF	61.5 (55-65)	65 (56-67)
Ginsini score	34 (20-60)	32 (20-58)
Multivessel disease, n (%)	487 (51.4)	19 (45.2)
Medical history and coronary risk factors		
Smoking, n (%)	629 (66.4)	5 (11.9)
BMI (kg/m^2)	28.09 (25.64-31.12)	28.38 (25.12-32.41)
Hypertension, n (%)	441 (46.5)	19 (45.2)
Diabetes mellitus, n (%)	203 (21.4)	15 (35.7)
Hyperlipidemia, n (%)	365 (38.5)	15 (35.7)
Family history of CAD, n (%)	92 (9.7)	7 (16.7)
Familial hypercholesterolemia, n (%)	24 (2.5)	3 (7.1)
Pre-admission Medications		
Antiplatelet drugs, n (%)	23 (2.4)	3 (7.1)
Antihypertensive drugs, n (%)	191 (20.1)	14 (33.3)
Hypoglycemic drugs, n (%)	76 (8.0)	8 (19.0)
Antilipidemic drugs, n (%)	42 (4.4)	2 (4.8)
Laboratory results		
Creatinine (μmol/L)	74.65 (66.45-83.65)	52.90 (47.13-58.05)
eGFR (mL/min/1.73m^2)	116.38 (105.31-122.32)	122.19 (116.38-126.25)
Fasting blood glucose (mmol/L)	5.36 (4.90-6.49)	5.39 (4.92-8.78)
Hemoglobin A1c (%)	5.6 (5.3-6.5)	5.7 (5.2-9.0)
TG (mmol/L)	1.89 (1.33-2.83)	1.2 (0.90-2.13)
Total cholesterol (mmol/L)	4.55 (3.64-5.46)	4.36 (3.80-5.78)
HDL-C (mmol/L)	0.89 (0.78-1.02)	1.00 (0.82-1.82)
LDL-C (mmol/L)	2.74 (2.06-3.59)	2.59 (2.12-3.81)
Uric acid (μmol/L)	410.20 (349.90-470.60)	309.8 (245.08-379.6)

AIP	0.33 (0.15-0.54)	0.13 (-0.07-0.37)
hs CRP (mg/L)	2.91 (0.98-8.60)	1.51 (0.59-8.88)

Notes: Data are expressed as medians with interquartile range for continuous variables with non-normal distribution and number (%) for categorical variables. BMI, body mass index; CAD, coronary artery disease; eGFR, estimated glomerular filtration rate; TG, triglyceride; HDL-C, high-density lipoprotein cholesterol; LDL-C, low-density lipoprotein cholesterol; AIP, atherogenic index of plasma; hs CRP, high-sensitivity C- reactive protein; LVEF, left ventricular ejection fraction.
